# Efficient *in silico* exploration of RNA interhelical conformations using Euler angles and WExplore

**DOI:** 10.1093/nar/gku799

**Published:** 2014-10-07

**Authors:** Alex Dickson, Anthony M. Mustoe, Loïc Salmon, Charles L. Brooks

**Affiliations:** 1 Department of Chemistry, University of Michigan, 930 N University, Ann Arbor, MI 48109, USA; 2 Department of Biophysics, University of Michigan, 930 N University, Ann Arbor, MI 48109, USA

## Abstract

HIV-1 TAR RNA is a two-helix bulge motif that plays a critical role in HIV viral replication and is an important drug target. However, efforts at designing TAR inhibitors have been challenged by its high degree of structural flexibility, which includes slow large-amplitude reorientations of its helices with respect to one another. Here, we use the recently introduced algorithm WExplore in combination with Euler angles to achieve unprecedented sampling of the TAR conformational ensemble. Our ensemble achieves similar agreement with experimental NMR data when compared with previous TAR computational studies, and is generated at a fraction of the computational cost. It clearly emerges from configuration space network analysis that the intermittent formation of the A22-U40 base pair acts as a reversible switch that enables sampling of interhelical conformations that would otherwise be topologically disallowed. We find that most previously determined ligand-bound structures are found in similar location in the network, and we use a sample-and-select approach to guide the construction of a set of novel conformations which can serve as the basis for future drug development efforts. Collectively, our findings demonstrate the utility of WExplore in combination with suitable order parameters as a method for exploring RNA conformational space.

## INTRODUCTION

The conformational flexibility of RNA molecules is increasingly recognized as critical to how RNAs carry out their functions (1). While this flexibility has been characterized experimentally, an atomic-level picture is in many cases lacking. Molecular dynamics (MD) simulation is a useful tool that can provide dynamical information at atomic resolution, but sampling of large amplitude motions is often hindered by high energy barriers. Even the simplest motions, such as stacking and unstacking of unpaired bases, can take place on timescales approaching hundreds of nanoseconds, making their characterization by standard MD methods difficult (2). As we seek a better understanding of RNA biology and attempt to rationally design RNA binders it is thus necessary to develop methods that make sampling such motions more accessible.

One of the most common and biologically important types of motion in RNA is interhelical dynamics (2,3). Complementary pairing of secondary structure base pairs results in highly stable A-form helical domains that are connected by flexible single-stranded and non-canonically paired internal loops, bulges and higher-order junction motifs. In the absence of stabilizing long-range tertiary interactions, helices can thus undergo large amplitude bending and twisting motions on microsecond timescales. Interhelical motions have been shown to be critical to biological function. In ribozymes, ‘docking’ and ‘undocking’ of the substrate helix into the catalytic active site facilitates substrate recognition and product release (4,5). In riboswitches, large-scale opening and closing motions of helical domains about higher-order junctions allow ligands to access otherwise buried binding sites (6), and during translation by the ribosome, interhelical dynamics of both tRNA and elements of the ribosomal RNA, like the L1 stalk, facilitate translocation (7,8).

**Figure 1.**
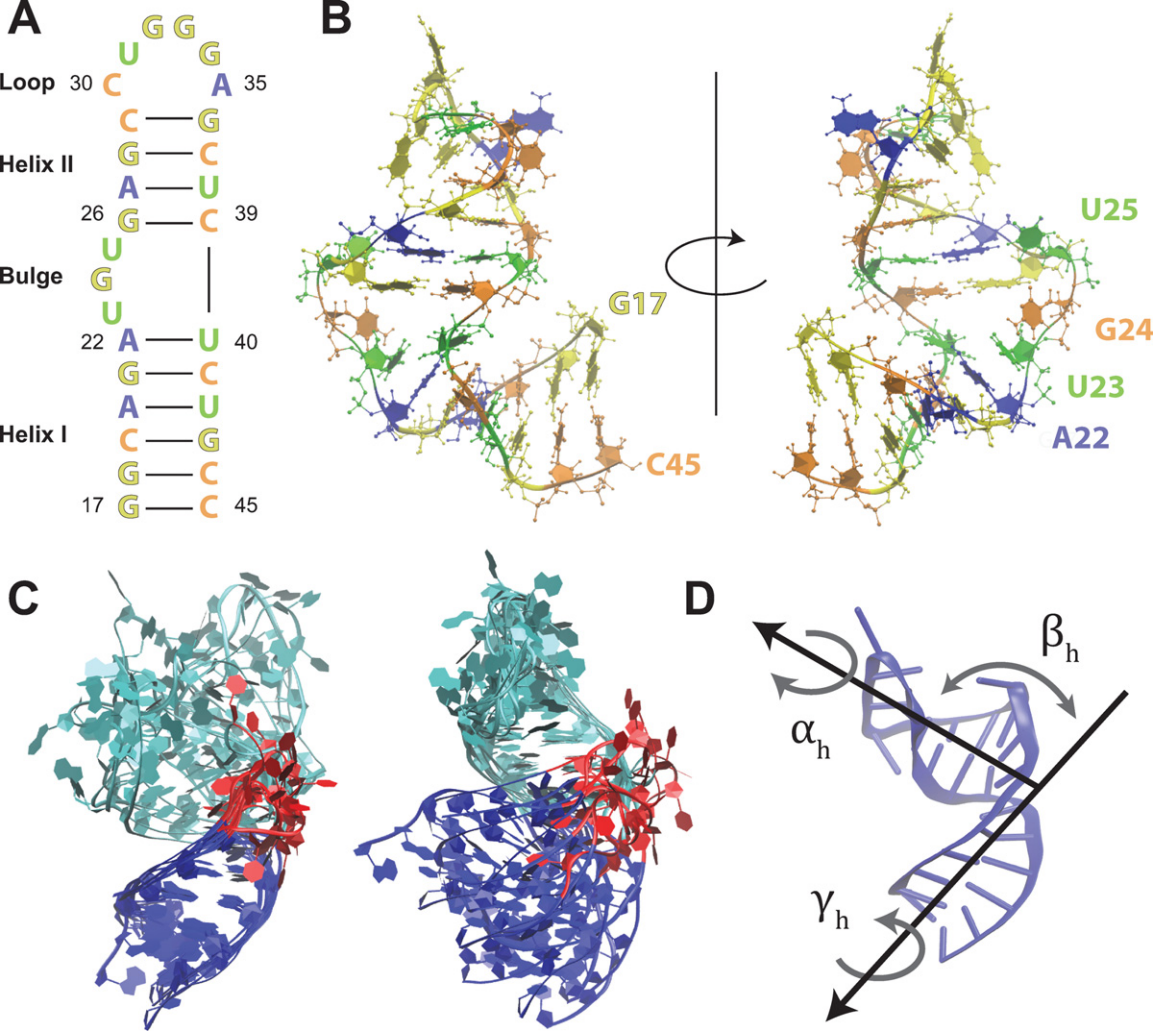
(A) The secondary structure of the TAR hairpin. Residues are color-coded to match the structures on the right. (B) HIV-1 TAR RNA is shown using cartoon and CPK representations. The structure shown is the first NMR model of PDBID 1ANR (52) after energy minimization. (C) Structures of ligand bound conformations (PDBIDs: 2L8H (23), 1ARJ (70)), 1QD3 (17), 2KX5 (22), 2KDQ (21), 1UUD (19), 1LVJ (18), 1UUI (19) and 1UTS (20)), aligned using Helix I (left) and Helix II (right). Helix I is colored in dark blue, Helix II in light blue and the bulge region is colored red. (D) A diagram showing the interhelical Euler angles α_*h*_, β_*h*_ and γ_*h*_. Color figure is available online.

A canonical example of interhelical dynamics is that exhibited by the trans-activating response (TAR) element of HIV-1 RNA (Figure 1). Early studies have identified that the two helical segments of TAR - connected by a three nucleotide bulge - adopt radically different conformations with respect to one another depending on environmental salt concentration or ligand binding (9). More recently, nuclear magnetic resonance (NMR) studies have shown that TAR samples these various global conformations at equilibrium on timescales varying from hundreds of nanoseconds to tens of microseconds (10,11). It has been shown that these long timescales can result from coupling to local motions, for instance, the formation and breakage of the A22-U40 base pair adjacent to the bulge has been linked to global motions, such as interhelical bending (12,13).

These studies have been largely motivated by the considerable importance of TAR as a drug target. Located at the beginning of the 5′ LTR (long terminal repeat), TAR plays a critical role in promoting efficient transcription of the downstream genome by binding the viral Tat protein and the host cofactor Cyclin T1 (14–16). As the TAR-Tat interaction provides a drug target that is unique to the virus, much work has been focused on the identification of small molecules that can inhibit this interaction (17–25).

The inherent flexibility of RNAs, such as TAR, is one of the major obstacles in designing RNA-targeted small molecules (26,27). To account for conformational heterogeneity of the binding site in TAR, conventional computational docking protocols have been modified to dock to entire ensembles rather than using a single binding site structure (28). Furthermore, there is also evidence that the structural heterogeneity of RNA can extend into the bound state (29,30), resulting in so-called fuzzy complexes (31). This motivates an ensemble-based drug targeting strategy that explicitly models the thermodynamic effects of flexibility (32). Although it has been shown that ensembles can be generated by knowledge-based structure prediction methods (33), approaches based on MD can provide both thermodynamic and kinetic information on the conformational ensembles of RNA molecules. One such approach has utilized NMR residual dipolar coupling (RDC) measurements and a sample-and-select (SAS) strategy to identify all-atom structures that match experimental data, chosen from a MD-generated pool (13,34). However, this requires a comprehensive initial pool from which to select structures and thus suffers from sampling challenges due to long equilibration timescales.

Although many methods exist to enhance sampling of transitions between two basins in MD simulations (35–41), a smaller set of methods can enhance sampling in an undirected fashion (42,43), often using a set of order parameters to define a sampling space (44–46). To guide sampling for RNA interhelical junctions it is natural to use Euler angles (α_*h*_, β_*h*_, γ_*h*_) as order parameters: these together provide a natural description of RNA interhelical conformation, with α_*h*_ and γ_*h*_ representing the twist of the two helices about their helical axes, and β_*h*_ the interhelical bend (47,48). As these variables are not differentiable as a function of atomic coordinates, sampling methods that apply biasing forces along order parameters cannot be used in this framework (44–46).

We recently introduced the algorithm WExplore that was specifically designed to build ensembles in an undirected fashion by enhancing sampling in a multidimensional order parameter space (49). It works by evolving a set of trajectories forward in time, and periodically uses cloning and merging steps to maximize heterogeneity in the trajectory set. As it does not require the use of biasing forces, we are able to employ Euler angles to define sampling regions. We generate an ensemble of configurations for HIV-1 TAR that shows better agreement to a set of experimental RDCs, while using a fraction of the computational cost when compared to a previous study that employed 8 μs MD simulations (13). We observe many novel interhelical conformations, including those with large values of total twist (α_*h*_ + γ_*h*_) and large interhelical bends (β_*h*_), and show that the intermittent formation of the A22-U40 base pair serves as a gatekeeper that must be broken to sample a large ensemble of highly twisted regions. Finally, we use a SAS procedure on the WExplore ensemble and in combination with conformations sampled by the prior Anton simulation to construct new, comprehensive configurational ensembles of TAR. Overall, our data indicate that WExplore combined with order parameters, such as Euler angles, provide a powerful method for undirected exploration of RNA conformational space.

## MATERIALS AND METHODS

### MD of TAR

The trajectory segments are run using CHARMM (50) through the OpenMM-CHARMM interface, with the CHARMM36 force field for nucleic acids (51). An explicit solvent of 9580 TIP3P water molecules is used, along with 28 sodium ions to neutralize the system. Periodic boundary conditions in a cubic box with sides of length 66.2 Å are employed to ensure that the molecule does not interact with its image, even when accounting for large amplitude interhelical motions. SHAKE is used to constrain the hydrogen atoms along their covalent bonds, with a tolerance of 10^−8^. Particle-mesh Ewald summation is used for non-bonded interactions with a van der Waals switching function that scales the non-bonded interactions to zero at 11 Å, starting from 8 Å.

The first model of PDBID 1ANR (52) is used as a starting structure. After solvation, the solvent and ions are minimized using 500 steps of steepest descent and 500 steps of the adopted basis Newton–Raphson method. The entire system is then minimized in the same way. The system is then heated, with harmonic restraints on the RNA, from 50 to 300 K over 100 ps, using 2 fs timesteps. The constraints are then gradually removed by decreasing their strength from 5 kcal/mol to zero in 10 stages, with 10 ps of dynamics run at each stage. Equilibration of the entire system without restraints is then allowed for 5 ns. The resulting structure is used as an initial condition for all 48 replicas in the WExplore method.

Dynamics are performed in a constant pressure ensemble with a barostat coupled to a reference pressure of 1 atm, and volume moves attempted every 25 steps. We use a group of four graphics processing units, each running an instance of CHARMM with OpenMM 5.0 (53) to run trajectories for the replicas. These trajectories are managed by a Perl script that implements the WExplore method outlined below.

An additional WExplore simulation is run where the A22 and U40 bases are forced to remain intact. This simulation follows the same protocol described above, except a harmonic restraint is applied to the distance between the N1 atom of A22 and the N3 atom of U40 with a minimum of 3.03 Å and a strength of 400 kcal/mol.

### Weighted ensemble (WE)

We first describe the original WE algorithm (36), which is the basis for the WExplore method (49). In the WE algorithm, sampling of low probability states is enhanced by defining a set of regions that span the space, and encouraging even sampling among the regions. Multiple copies of the system (replicas) are run in parallel, and each is given a weight with which it contributes to statistical averages. Periodically throughout the simulation, the number of replicas in each region is kept constant by merging replicas in overrepresented regions, and cloning replicas in underrepresented regions. When a replica is cloned, its weight is split up evenly among the clones. When two replicas *A* and *B*, with weights *w*_*A*_ and *w*_*B*_, are merged, the resulting replica has weight *w*_*A*_ + *w*_*B*_, and takes on the configuration of replica *A* with probability *w*_*A*_/(*w*_*A*_ + *w*_*B*_), and otherwise takes on the configuration of replica *B*. High-weight replicas are also broken up by cloning, and low-weight replicas are merged, to encourage replicas in the same region to have approximately equal weights.

In the 1D WE simulations performed here for comparison, the order parameter used to define the regions is the magnitude of the single-axis rotation between the current set of interhelical angles (α_*h*_, β_*h*_, γ_*h*_) (47,48), and the interhelical angles of the NMR structure used to initialize the simulation. Five regions are defined along this order parameter with a spacing of 20°.

### WExplore: sampling using hierarchical regions

Recently, we introduced an extension to WE that allows for efficient application to high-dimensional spaces, where the number of walkers can be far less than the number of regions. The method has been described in detail in previous work (49). Briefly, WExplore uses a set of nested, hierarchical regions to balance computational effort using cloning and merging steps. The top level regions are very large, and are tiled by smaller regions, which are themselves tiled by even smaller regions, and so on. This allows for replica balancing across multiple length scales: a balancing procedure is first performed at the top level of the hierarchy, and then at each lower level, which prioritizes balancing computational effort among regions that are the farthest apart.

We use (α_*h*_,β_*h*_,γ_*h*_) angles to define the sampling regions using the distances of single-axis rotations. Regions are defined dynamically over the course of the simulation, and do not require any *a priori* structural information. We use a four-level sampling hierarchy with region sizes of 80°, 50°, 30° and 20°, and a maximum number of images of 10 at each sampling resolution, for a theoretical maximum of 10 000 regions. In practice, only five top-level regions, and 483 regions total, are found at the end of 680 sampling cycles.

Each sampling cycle consists of all 48 walkers running for 20 ps, followed by region assignment and cloning and merging steps. The aggregate simulation time of the WExplore simulation after 680 cycles is then 652.8 ns. For prediction of RDCs and chemical shifts, an ensemble of 10 000 walkers is generated by recording the structures of all the walkers at the end of each cycle, and using the 10 000 most recent structures. This is akin to using the last 31% of the simulation. Each structure is given a weight by diagonalizing the matrix of region-to-region crossing statistics as done previously (49,54,55). We note that although improved weight estimates could be achieved by using these weights to restart a new WExplore simulation and then waiting for these weights to equilibrate (as in Dickson and Brooks (49)), this would have dramatically increased computational cost, and was unnecessary since we eventually rely on a SAS framework to generate our ensembles.

### Computation of Euler angles

Interhelical (α_*h*_,β_*h*_,γ_*h*_) angles are computed essentially as previously described (48). Briefly, we align the lower helix of TAR to the lower helix of a reference frame consisting of two coaxially stacked idealized A-form helices. We then compute the rotation matrix needed to bring the upper TAR helix into alignment with the upper reference frame helix and deconvolute this rotation into Euler angles. To minimize computational time aligning TAR helices to the idealized reference helix, only C1′ and P atoms of each helical residue are used. The A22-U40 base pair is excluded from reference helix alignments given its known instability, with the H1 helix defined by residues 19–21 and 41–43 and the H2 helix defined by residues 26–28 and 37–39. Each TAR helix is required to align to the idealized reference helix with root mean squared distance (RMSD) < 2 Å. Conformations with one or more helices >2 Å from the idealized reference are excluded from cloning and merging steps in the WExplore algorithm until they achieve RMSD < 2 Å for both helical segments.

### Comparison to the topologically allowed set

Comparisons to the set of topologically allowed interhelical conformations are performed using the Euler angles that define each region of the configuration space network (see section ‘Configuration space network analysis’). By excluding the A22-U40 pair, the WExplore angles are computed assuming an S_4_S_1_ internal loop topology and are thus twisted relative to the set of Euler angles that would be topologically allowed for a S_3_S_0_ bulge topology (47,56). To correct for this twist, the WExplore angles are transformed by multiplying the rotation matrix defined by the WExplore Euler angles (*R*_WE_) by the rotation matrix defined by the Euler angles 78.15°, 0.14° and −45.46° (*R*_C_). *R*_C_ effectively rotates the reference helix alignment of the TAR lower helix up one base pair to the position it would have had if aligned using a S_3_S_0_ junction topology. The resultant matrix *R*_WE_*R*_C_ is then deconvoluted into corrected angles (α_*h*_,β_*h*_,γ_*h*_)_C_. The corrected angles are then compared against the set of topologically allowed angles for three nucleotide bulges determined from simulations of the TOPRNA coarse-grained model (57). WExplore angles are counted as topologically allowed if they are within a 10° single axis rotation distance of a topologically allowed angle.

### Construction of configuration space networks

We build the network graphs using the program Gephi (58), following the protocol in our previous work (49,59,60). The states used to build the graphs correspond to the lowest level (i.e. 20°) of the hierarchically organized set of images used in the WExplore algorithm during sampling. The sizes of the nodes are proportional to their statistical populations after weight balancing, however, for each graph we use a minimum node size that is 30 times smaller than the size of the largest node, due to visualization constraints. The orientation of the nodes is obtained using a force minimization algorithm built into Gephi (ForceAtlas), which introduces a repulsive force between all nodes, but attracts nodes that are linked together with a force that is proportional to the weights of the links, which are determined as follows. First, weights of directed links with values between 1 and 1000 are determined as *w*_*ij*_ = 1000*p*_*ij*_, where *p*_*ij*_ is the transition probability from *i* to *j*. Weights of undirected links are then determined as the average of the two directed links.

### Comparison of simulated ensembles with NMR data

The detailed description of the SAS procedure and experimental data measurements can be found in the literature (13). Briefly, four different sets of RDCs measured in Pf1 phage are used. Each set of data corresponds to a differentially elongated construct of HIV-1 TAR presenting a UUCG apical loop: E0 (61) corresponds to the non-elongated TAR construct, EI-22 (62) and EI-3 (63) to two constructs where the lower helix was elongated by 22 or 3 base pairs and EII-22 (62) to a construct in which the upper helix was elongated by 22 base pairs. The data set includes a variety of one-bond RDCs spanning the sugars (C1′-H1′, C2′-H2′, C3′-H3′ and C4′-H4′) and the bases (C2-H2, C5-H5, C6-H6, C8-H8, C5-C6, N1-H1 and N3-H3). Note that 44, 45, 36 and 38 RDCs are respectively used in the E0, EI-22, EII-22 and EI-3 TAR data sets. RDCs from conformations sampled in simulation are computed after appropriately elongating the molecule *in silico* (13,64) using the structure-based RDC prediction software PALES (65). In the RDC prediction procedure, an overall scaling factor is used for each construct to optimize the agreement with experimental data.

During selection, the conformations are assumed to be equally probable, and repeated conformers are not allowed. The selection procedure starts from a randomly selected subset of conformation of the desired size (here 20) and at each step we randomly substitute one of the conformers by another from the pool, following a Metropolis Monte Carlo algorithm as described previously (13).

All chemical shifts, including H1′, H2, H5, H6 and H8 are computed using NUCHEMICS (66). A total of 48 chemical shifts are used, spanning both the helices and the bulge. The terminal base pairs G17-C45 and C29-G36 are excluded (13).

The field-induced RDCs (fRDCs) are computed using previously described protocols (13,67).

## RESULTS

### Comparison of sampling ranges in Euler space

It has been previously shown (13,34,48,56,68) that the large-scale interhelical dynamics of two-way junctions can be well described by three Euler angles: α_*h*_, which describes the relative twist of the lower helix about its helical axis; γ_*h*_, which similarly describes the twist of the upper helix and β_*h*_, which describes the interhelical bend angle between the two helices. We used these three angles to define a sampling space for the WExplore algorithm in order to broadly sample the interhelical orientations of HIV-1 TAR in an undirected manner. We quantify the breadth of sampling by counting cubic elements (with side lengths of 10°) of Euler space that are visited by WExplore, and compare this to conventional simulation and 1D WE simulations of equivalent computational cost. The WExplore algorithm uses 48 trajectories in parallel that are periodically managed by cloning and merging operations that reduce redundancy in the trajectory set; this is described in detail in previous work (49). Conventional sampling results are obtained by switching off the cloning and merging operations in the WExplore algorithm, which results in 48 continuous trajectories. 1D WE simulations use regions defined along a single reaction coordinate, which is the distance of the single axis rotation in Euler space to the initial NMR structure (see Materials and Methods).

**Figure 2.**
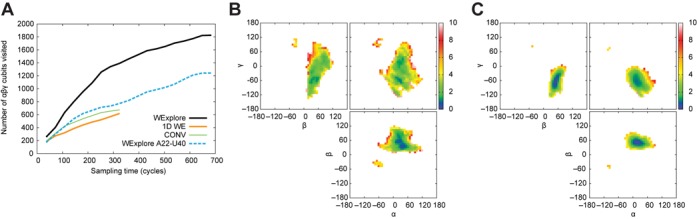
A comparison of sampling in Euler angle space. (A) A comparison of the volume of Euler angle space sampled by three methods: WExplore, 1D WE, and conventional sampling (see Materials and Methods section for details). The dashed blue line shows the sampling of a separate WExplore simulation with a harmonic restraint between bases A22 and U40. An (α_*h*_, β_*h*_, γ_*h*_) cubit is defined as a cube in Euler angle space with edge lengths of 10°. Projections of sampling distributions are shown on α_*h*_: β_*h*_, α_*h*_: γ_*h*_ and β_*h*_: γ_*h*_ planes for WExplore (B) and conventional sampling (C). For comparison, (B) and (C) both show sampling distributions after 330 cycles (6.6 ns per replica), although the WExplore simulation is continued to 680 cycles (13.6 ns per replica). The color bar in each panel shows the free energy in *kT* . Color figure is available online.

Figure 2A shows that WExplore samples over twice as much volume as 1D WE or conventional sampling at an equivalent sampling time. This is also visualized using 2D projections onto α_*h*_: β_*h*_, α_*h*_: γ_*h*_ and β_*h*_: γ_*h*_ planes (Figure 2B and C). Supplementary Figure S1 compares the same set of 2D projections with projections computed from a previous study using 8 μs of straightforward simulations on the Anton supercomputer, which employed the same force field and initialization protocol used in this work. Notably, although the WExplore simulation used about 8% of the simulation time of the Anton simulation (0.7 versus 8.2 μs), we sample many interhelical conformations that were not seen in the Anton study. These new conformations are characterized by either large interhelical bends (β_*h*_ ≥ 120°), or large twists (α_*h*_ ≥ 60°, γ_*h*_ ≥ 60°). We discuss these new states at length below.

However, there are also states not sampled by WExplore which were found previously by the Anton simulations. Notably, low interhelical bend states with coaxial stacking of the helices across the junction (β_*h*_ < 20°) are not sampled in our simulations. Both NMR and combined NMR-MD studies of TAR have shown that the bulge residue U23 is typically stacked on top of A22, which is mutually exclusive with interhelical stacking (13). In our simulations, we never observe unstacking of U23 (Supplementary Figure S2), which further supports the coincidence of U23 base-flipping with sampling of near-coaxial states. That U23 and A22 remain stacked throughout the simulation likely stems from insufficient sampling of degrees of freedom orthogonal to the Euler angles. Each trajectory in the WExplore pool is started at the same NMR model structure, and at the end of the simulation each of the 48 trajectories in the pool has been run for a total of 14 ns. This length of time is thus likely insufficient to sample A22-U23 unstacking.

### Configuration space network analysis

Motivated by the discovery of new interhelical conformations, we seek to visualize how these states fit into the conformational ensemble of HIV-1 TAR. To this end, we create configuration space networks where each node represents a unique interhelical configuration, and the connections (edges) between the nodes show transitions between configurations. The nodes used here are the smallest sampling regions in the hierarchy used by the WExplore algorithm. The arrangement of the nodes in the network is determined using a force minimization algorithm, where nodes are naturally repelled from each other, but nodes that are connected are pulled closer together. Network plots can thus reveal features of the global dynamics of the free energy landscape, in that nodes that are close together are likely to interconvert faster than nodes that are far apart. Details on the construction of the network plots is given in Materials and Methods. Information for a representative structure for each node is given as Supplementary Worksheet 1, and a network with labels for each node is given as Supplementary Figure S6. The representative structures are available for download from http://brooks.chem.lsa.umich.edu/data.

#### Two partitions: open and closed

**Figure 3.**
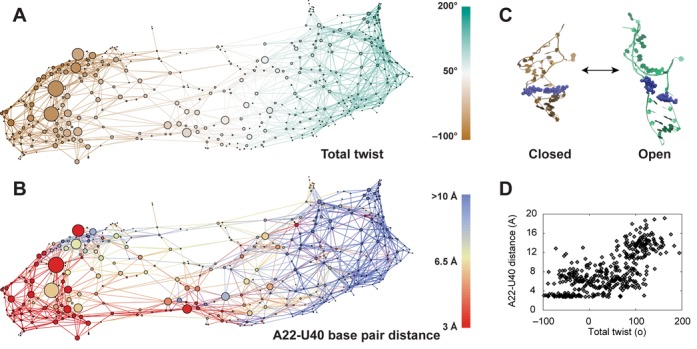
The physical basis of the observed partitioning of the configuration space network of TAR. (A) The configuration space network of TAR colored to show the total twist (α_*h*_ + γ_*h*_) of each node. Each node in the network represents a given (α_*h*_, β_*h*_, γ_*h*_) conformation and the links between nodes show the transitions between conformations. (B) The nodes are colored to show the A22-U40 base pair distance within each conformation. All nodes with distances greater than 10 Å are colored blue. (C) Representative conformations of the two network partitions, which we label ‘closed’ and ‘open’ for the left and right partition, respectively. The A22 and U40 bases are shown in van der Waals representation. (D) Scatter plot showing the A22-U40 distance versus the total twist for each node. Color figure is available online.

Analysis of the TAR CSN shows a partitioning of the network into two groups of states (Figure 3A), which can be clearly demarcated by the total twist of the system (α_*h*_ + γ_*h*_). On the left side of the network, negative twist results in a globally compact configuration that we refer to as the ‘closed’ partition. On the right side, large positive twist values result in a set of extended structures that we refer to as the ‘open’ partition. Representative structures of both partitions are shown in Figure 3C.

There is a significant correlation of the total twist with the distance between the A22 and U40 bases (*d*_22 − 40_). When this base pair is formed (*d*_22 − 40_ < 4 Å) the system is largely confined to low values of total twist, below 50° (Figure 3D). Breakage of this base pair then allows for the sampling of the open partition. To confirm that A22-U40 breakage is required to sample these states we run a WExplore simulation with an added distance restraint applied to the bases of A22 and U40. This constrained simulation has reduced sampling along the Euler angles and does not sample highly twisted structures (Figure 2A, Supplementary Figure S1).

**Figure 4.**
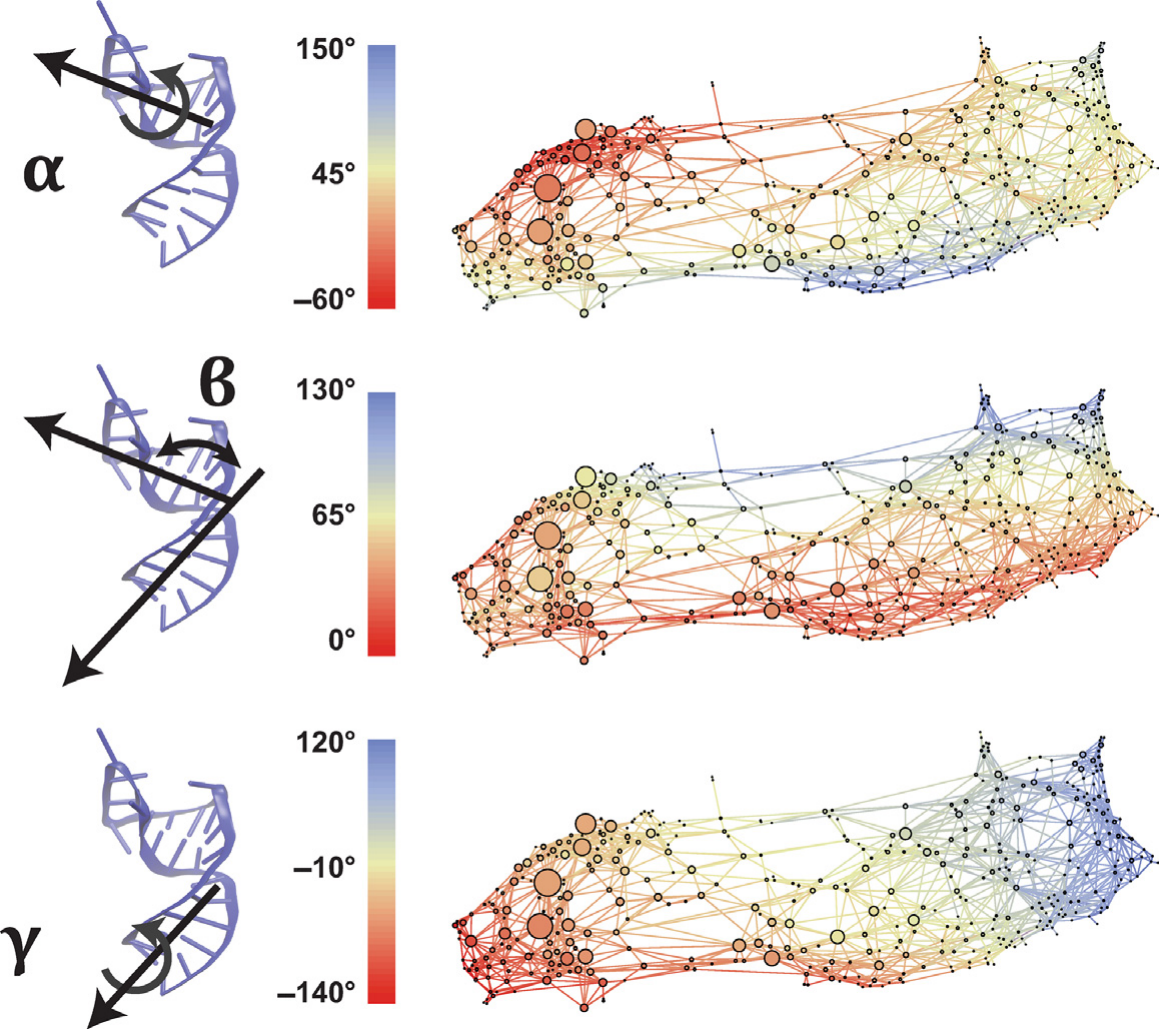
Visualization of interhelical conformations. Node coloring shows the sampling along the α_*h*_ (top), β_*h*_ (middle) and γ_*h*_ (bottom) interhelical variables. In contrast to Figure 2, here we enforce that β > 0 through the symmetry transformation (α, β, γ) → (α + 180°, −β, γ + 180°), for better visualization. Color figure is available online.

From network plots showing α_*h*_, β_*h*_ and γ_*h*_ separately (Figure 4) we see that the two interhelical twist variables α_*h*_ and γ_*h*_ are anti-correlated, which has been shown previously (47,56), and that this anti-correlation holds in both the open and closed partitions. The β_*h*_ map (middle) reveals highly bent states (|β_*h*_| > 100°) in both the open and closed partitions. Cross-referencing with Figure 3B reveals that these large interhelical bends are facilitated by the broken A22-U40 base pair.

**Figure 5.**
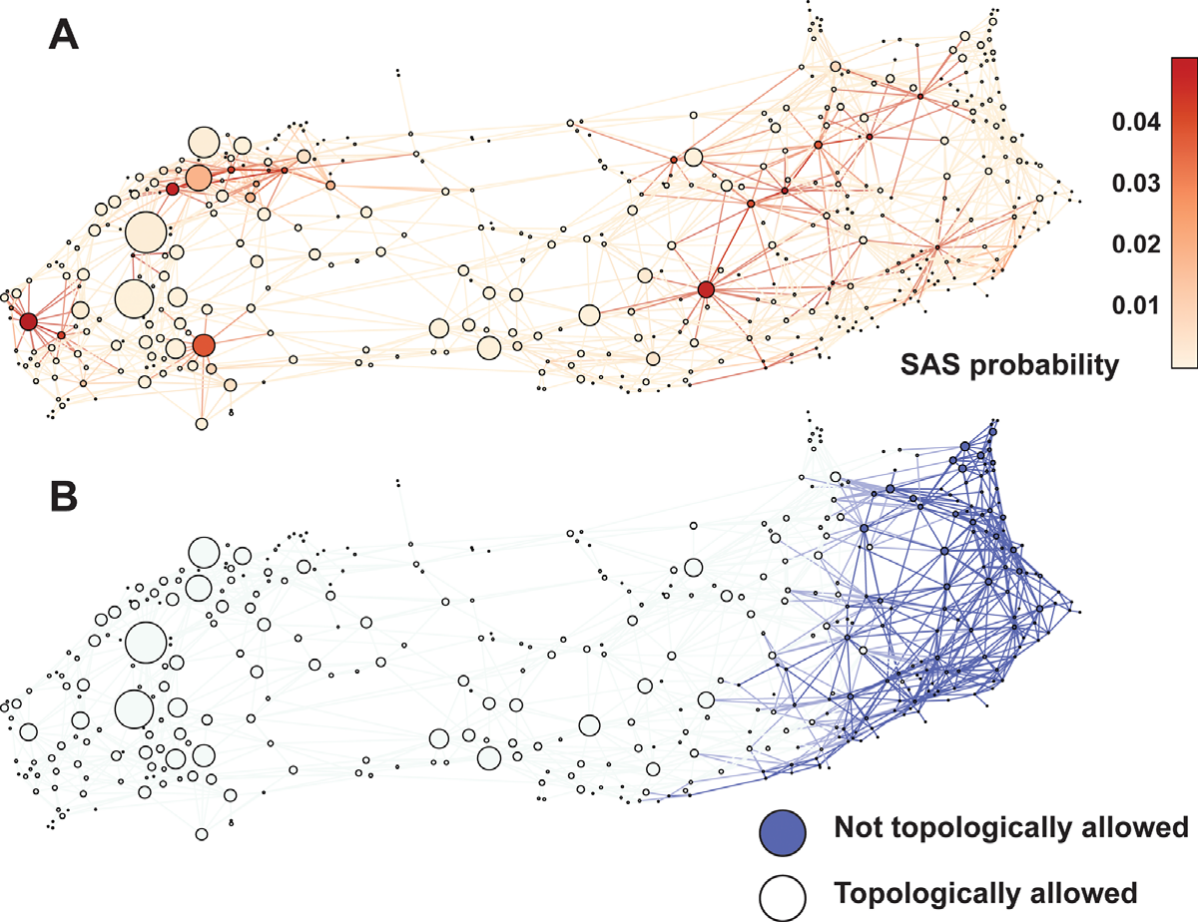
(A) Probability of a node to be selected by the RDC-based SAS procedure. (B) Detection of states that are not topologically allowed assuming a 3-0 bulge topology. Color figure is available online.

Previous studies from our labs have shown that steric and connectivity constraints imposed by the secondary structure of junctions strongly limit the set of accessible interhelical conformations of an RNA (47,56,57). While these ‘topological’ constraints normally limit 3-nucleotide bulges to only 15% of the Euler angle space, we find that distortions of the junction-closing base pairs could partially alleviate these constraints, expanding the set of allowed conformations (56). We investigate whether breaking of the A22-U40 pair correlates with sampling of otherwise topologically inaccessible states. Comparison to the previously determined set of allowed states shows that 133 of the 438 nodes in the network would be topologically inaccessible, all corresponding to states with broken or highly distorted A22-U40 pairs (Figures 3B and 5B). Figure 5 shows that some of these otherwise topologically disallowed states are strongly selected when a SAS procedure is used to select a subensemble of conformations that show maximal agreement with NMR RDCs (see ‘Sample and select’ below), suggesting that these states play an important role in the natural dynamics of TAR. We also find that all of the states sampled by the A22-U40 restrained simulation fall within the ensemble of topologically allowed 3 nt bulge states. These observations, together with the knowledge that the A22-U40 base pair is known to be unstable in solution (61,69), point to the role of A22-U40 as a reversible entropic gate that can enlarge or contract the space of accessible interhelical configurations.

#### Mapping known ligand-bound structures

**Figure 6.**
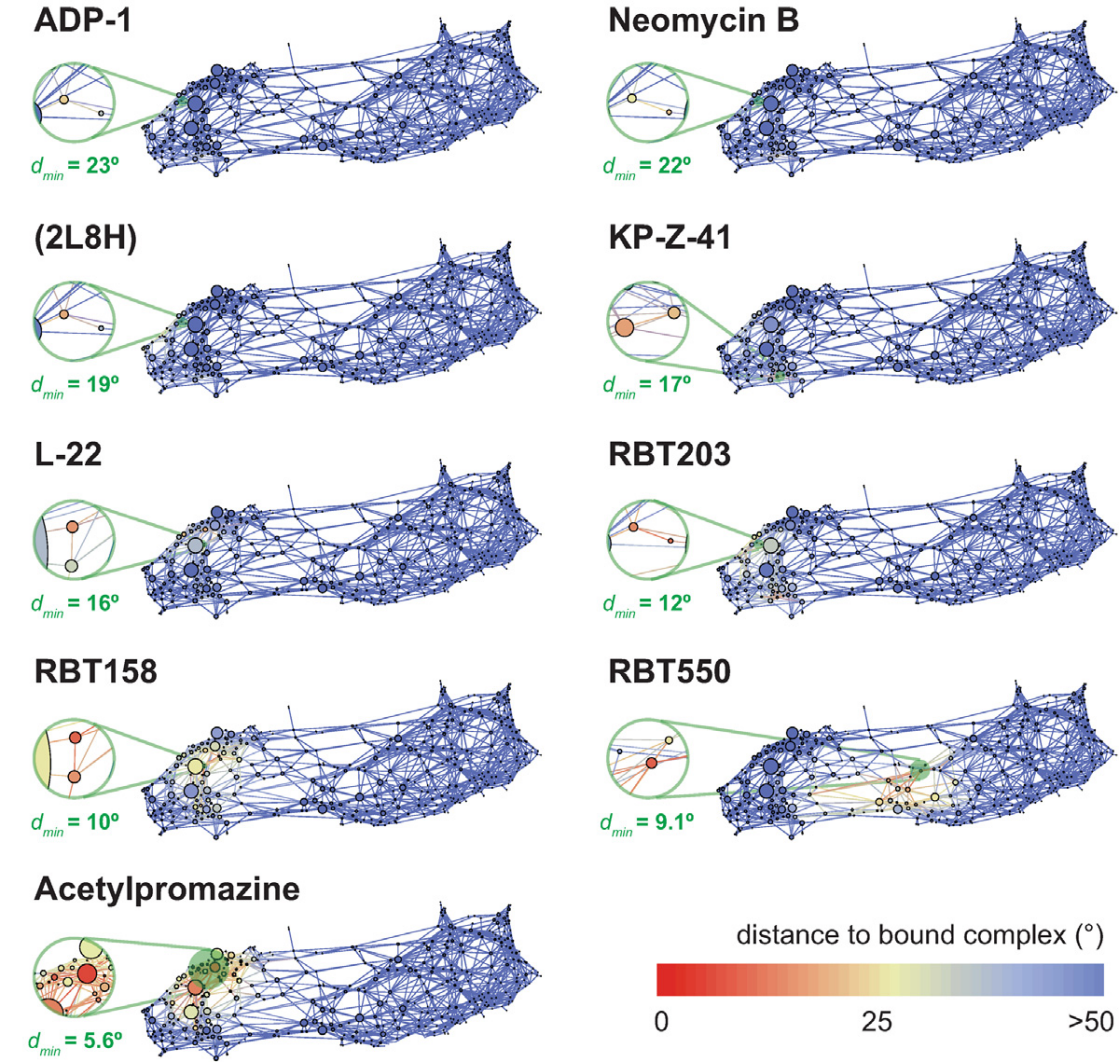
Mapping ligand-bound structures on the TAR CSN. For each ligand-bound structure, we show a CSN for TAR with the node colorings indicating the distance in Euler angle space from each node to the ligand-bound structure. For NMR structures with multiple models, we plot the distance to the first model in the structure. Nodes that are greater than 50° are colored blue, and red nodes reveal structures that are close to the ligand-bound structure. Each plot is labeled by the name of the inhibitor molecule, except for PDBID:2L8H (23), which shows the bound structure of arginine 4-methoxy-naphthylamide. The PDB indices for the other bound structures are as follows: ADP-1 (1ARJ (70)), Neomycin B (1QD3 (17)), KP-Z-41 (2KX5 (22)), L-22 (2KDQ (21)), RBT203 (1UUD (19)), Acetylpromazine (1LVJ (18)), RBT158 (1UUI (19)), RBT550 (1UTS (20)). Insets are shown that focus on the nodes that are closest to the bound structure, and the number below each shows the closest distance to the bound structure. The plots are sorted by the overall closest distance, and range from 23° for ADP-1 to 5.6° for Acetylpromazine. The insets are connected to transparent green circles in each network that show the area prior to magnification. Color figure is available online.

HIV-1 TAR RNA plays a critical role in the infectivity of HIV; after it is transcribed by RNA Pol II, it binds the viral Tat and host Cyclin T1 proteins that are vital for promoting efficient transcription of the downstream HIV genome (14–16). As such, much work has focused on developing inhibitors of the interaction between TAR and Tat (17–23,70). In Figure 6, we map a set of known structures of TAR with inhibitors bound. For the four bound complexes shown at the top of Figure 6, there only exist a few states that are close to the bound structure, and they are not significantly populated as determined by the sampling in WExplore. Although this might suggest a binding mechanism of induced fit, an alternative explanation is that these conformations are significantly populated in the dynamics of TAR, but are simply not populated here due to insufficient sampling. Consistent with our discussion above, these four complexes have near-coaxial interhelical conformations (β_*h*_ = 3.8°, 7.2°, 3.0° and 7.5°, for ADP-1, Neomycin B, 2L8H and KP-Z-41, respectively). Note that we are using a Euler distance of 20° to define the smallest regions in WExplore, so a distance of greater than 20° can be considered to be not sampled by our ensemble.

Several interesting observations arise from analysis of the ligand-bound TAR structures that are sampled by WExplore. Acetylpromazine-bound TAR has the lowest distance of all ligand-bound structures to WExplore sampled regions, and is close (<20°) to many well-populated regions. This inhibitor is unique as it was identified through computational screening against a solution NMR structure of TAR (18). It is not surprising, then, that the bound structure is close to high probability regions of the TAR ensemble. Notably, all ligand-bound structures except for one (RBT550) are closest to states in the closed partition. RBT550 was designed following an alternative drug design strategy, where a non-functional conformation of TAR is targeted for drug binding (20). Our results suggest that this conformation exists within the natural ensemble of interhelical conformations, in the transition region between the open and closed partitions of the network.

Generally, the open partition provides a large number of potential drug targets that have not yet been explored, and are promising starting points for the discovery of novel TAR inhibitors. A first pass drug discovery approach could focus on states found here in the open partition which also have a high SAS probability (Figure 5A). As protein-bound RNA molecules can also be highly dynamic (30,71), an alternative approach would be to target instead an entire ensemble of RNA conformations that are fast interconverting, such as the entire set of structures in the ‘open’ partition. This would also result in a lower entropic binding cost, and is consistent with recent observations that so-called ‘loose binders’ of TAR are stronger Tat competitors than tight ones (29).

### Comparison with experimental NMR data

NMR RDCs report on the alignment of a bond vector connecting two nuclear spins to the external magnetic field of the spectrometer. As the interhelical dynamics in TAR can significantly change the alignment of many bond vectors concurrently, RDCs are good reporters for measuring large-scale interhelical dynamics. RDCs have been previously measured on TAR and different elongated TAR constructs, allowing for significantly orthogonal RDC data sets (13,34,62,72). Using the program PALES (73), and following previous work (13,34), we use our simulated ensemble to predict RDC values for both TAR and its elongated constructs.

**Figure 7.**
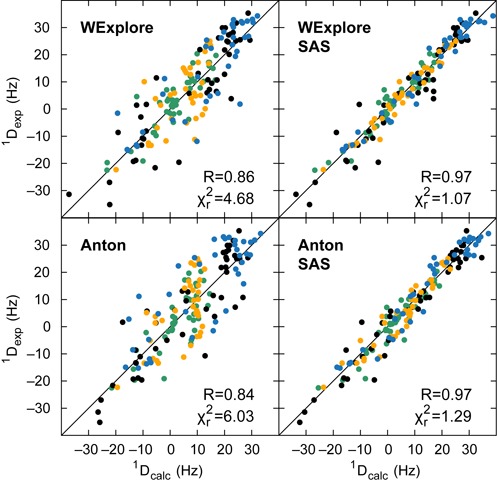
Agreement of our simulated ensemble with experimental RDC values of HIV-1 TAR and three elongated constructs. Each dot compares an experimental RDC value to its value predicted from the simulated ensemble. The dot colors are as follows: green is non-elongated TAR; black is EI-22, where Helix I is elongated by 22 base pairs; yellow is EII-22, where Helix II is elongated by 22 base pairs and blue is EI-3, where Helix I is elongated by three base pairs. The top-left and bottom-left panels show the agreement of the raw ensembles predicted by WExplore and Anton with experiment. The top-right and bottom-right panels show the experimental agreement of ensembles generated by SAS using the WExplore and Anton simulation as starting ensembles. Color figure is available online.

The WExplore ensemble results fit to the experimental results with $\chi ^2_r = 4.68$ and *R* = 0.86, which compare favorably to $\chi ^2_r = 6.03$ and *R* = 0.84 measured from Anton simulation previously (Figure 7). That we obtain somewhat better agreement with experimental RDC values using WExplore is significant, as it suggests that the addition of the large interhelical bend states, and heavily twisted states are more important than the loss of the near-coaxial, β_*h*_ = 0 states for reproducing RDCs. This is consistent with analysis based on ultra-fast time resolved fluorescence spectroscopy, which suggests that the population of the coaxially stacked state for HIV-1 TAR is only populated to a level of <5% (25). fRDCs, however, show the reverse trend with an RMSD of 1.17 Hz for WExplore and 0.90 Hz for Anton, possibly due to the different sensitivity of the alignment to the interhelical orientations.

In contrast to RDCs, chemical shifts report predominantly on local motions, and are especially useful in this context to examine the dynamics of the bulge and loop nucleotides. Supplementary Figure S3 shows the agreement of the chemical shifts predicted by the WExplore ensemble, which overall has slightly worse agreement (RMSD = 0.22 ppm) than that obtained from the Anton trajectory (RMSD = 0.20 ppm). WExplore shows large differences for chemical shifts in the bulge region, especially for the H1′ atom of base C24, and the H8 atom of base U25. This is likely due to the above discussed lack of sampling of flipped-out states for U23, which causes us to not properly model the full ensemble of bulge configurations (Supplementary Figure S2). Interestingly, flipped-in and flipped-out conformations are both sampled for C24 itself. U25 is found to be highly dynamic and mostly solvent exposed, and it does not sample favorable interactions between U25 and C24, which could also be the source of the disagreements in the C24 and U25 chemical shifts.

There are also chemical shifts where WExplore does significantly better than Anton. Rather than point to the enhanced local sampling of WExplore in these regions, this is likely due to unphysical configurations reached by Anton at long times. For instance, the terminal G17-C45 base pair is dissolved for some parts of the Anton simulation, which disrupts stacking interactions between G17 and G18. As WExplore uses a much shorter per-trajectory simulation time than Anton, it escapes from possible structural deterioration at long timescales related to imperfections in the force field, which could explain the difference in the accuracy of the G18 H1′ chemical shift.

### Sample and select

Prior MD studies of TAR, including the Anton simulation, used a SAS approach to select subensembles from the generated conformational pool that better reproduced experimental RDC values and thus better represent the true conformational landscape of TAR. We explored whether the enhanced sampling in our WExplore simulations would be useful for such SAS applications. As there is stochasticity built into the SAS process, both in choosing the initial ensemble and in the individual swap decisions, it can be repeated multiple times to get a probability that a given node is chosen during SAS, which is shown in Figure 5A. Notably, nodes that are well represented in the SAS ensemble are distributed relatively evenly between the open and closed partitions in the network, which suggests that both partitions are important to the natural dynamics of TAR. We find that although some high-weight states are not selected, there is an enrichment of high probability states in the SAS ensemble (Supplementary Figure S4). This is significant, as the procedure for picking a new conformer in the SAS procedure is independent from its weight in the trajectory.

The WExplore SAS ensemble, as expected, has significantly improved agreement with NMR measurements (Table 1). The $\chi ^2_r$ fit with experimental RDCs decreases from 4.68 to 1.07, which can be compared to the prior results of Salmon *et al.* (13), where $\chi ^2_r$ decreases from 6.03 to 1.29. This trend is confirmed by passive analysis where one of the four sets of RDCs is excluded from the selection procedure, or by randomly excluding individual RDCs from all four RDC sets from the selection procedure. SAS improves WExplore reproduction of the passive data from $\chi ^2_r=3.46$ to $\chi ^2_r=2.36$, compared to an improvement of $\chi ^2_r=3.49$ to $\chi ^2_r=2.85$ when SAS is used on the Anton ensemble. The SAS ensemble also better reproduces other experimental data not used in the selection: the RMSD of chemical shifts decreases from 0.22 to 0.19 ppm upon selection, and fRDC RMSD decreases from 1.17 to 0.98 Hz. As a comparison, the Anton-based selection leads to an improvement of chemical shift RMSD from 0.20 to 0.17 ppm and from 0.90 to 0.59 Hz for fRDCs. Thus, the trend discussed above holds: WExplore slightly better reproduces RDCs measured in Pf1 phage, while the Anton trajectory better reproduces chemical shifts and fRDCs. The better WExplore reproduction of phage RDCs might be correlated with the sampling of highly bent and twisted states by WExplore, while the differences in chemical shifts and fRDCs may arise from the less complete description of the bulge dynamics, especially regarding the nearly coaxial states.

**Table 1.** Agreement with experimental NMR measurements

		WExplore		Anton		Joint	
		raw	SAS	raw	SAS	raw	SAS
Active	RDC $\chi ^2_r$	4.68	1.07	6.03	1.29	3.68	0.90
	RDC *R*	0.86	0.97	0.84	0.97	0.73	0.98

Passive	RDC set $\chi ^2_r$		3.46		3.49		3.32
	RDC set *R*		0.90		0.90		0.91
	RDC random $\chi ^2_r$		2.36		2.85		2.39
	RDC random *R*		0.94		0.93		0.95
	fRDC RMSD (Hz)	1.17	0.98	0.90	0.59	1.00	0.87
	CS RMSD (ppm)	0.22	0.19	0.20	0.17	0.18	0.19

The Anton results are as reported by Salmon *et al.* (13), and the joint ensemble is a 50/50 mix of the WExplore and Anton ensembles. ‘Active’ statistics indicate values computed from data optimized in the selection procedure, while ‘passive’ statistics are computed from values left out of the selection and can be used as consistency checks. The passive ‘RDC set’ refers to the average agreement obtained for a given set of RDC measurements that are left out of the selection (either E0, EI-22, EII-22 or EI-3), and ‘RDC random’ refers to the agreement obtained for a randomly chosen set of RDCs that are omitted from the selection process.

As both the WExplore and Anton simulations have unique strengths and weaknesses, we also build a joint WExplore/Anton ensemble and examine its agreement with experimental data. The agreement with RDC values improves upon the agreement obtained by WExplore: in the raw ensemble the fitting parameters are *R* = 0.90 and $\chi ^2_r = 3.68$, and these improve upon SAS to *R* = 0.98 and $\chi ^2_r = 0.73$. The joint SAS ensemble reproduces passive RDCs with $\chi ^2_r=3.32$ and *R* = 0.91 for given sets that are left out of the selection process, and $\chi ^2_r=2.39$ and *R* = 0.95 for randomly removed RDCs. Chemical shifts agree to within 0.18 ppm pre-selection and 0.19 ppm post-selection, and fRDCs improve from 1.00 to within 0.87 Hz after selection. In summary, the newly obtained ensemble provides a better reproduction of both active and passive RDCs, and the agreement of chemical shifts and fRDCs is in between the individual WExplore and Anton ensembles. Supplementary Figure S5 shows the probability of selecting given conformers projected onto the α_*h*_: β_*h*_, α_*h*_: γ_*h*_ and β_*h*_: γ_*h*_ subplanes for WExplore, Anton and the joint ensemble. This reveals that both the near-coaxial states sampled by Anton and the high-twist and high-bend states sampled by WExplore are selected from this joint ensemble, implying that both sets of conformations are important to describe the natural dynamics of TAR.

## DISCUSSION

Efforts to target the TAR-Tat interaction to block HIV viral replication have been complicated by the dynamic nature of the TAR molecule. Here, we efficiently built a broad ensemble of TAR conformations using WExplore, and identified new states with high total twists and large interhelical bends that are consistent with experimental NMR RDCs. Notably, the broader conformational sampling obtained by WExplore led to better *a priori* agreement with experimental NMR RDC values compared to a previous 8 μs simulation run on the Anton supercomputer (13). This advantage was maintained when a SAS procedure was used to build subensembles optimized for their experimental agreement from each simulation. These results indicate that slow interhelical dynamics are likely a primary limiting factor in achieving better agreement between simulation and experiment.

The improved experimental agreement of WExplore is consistent with other studies showing that longer simulation timescales yield better agreement with experiment. The above discussed 8 μs Anton simulation of TAR yielded significantly better experimental agreement than a shorter 80 ns simulation (13,34). We also recently showed that a coarse-grained model that neglects both electrostatics and attractive interactions, yet which is able to exhaustively sample TAR interhelical space, achieves even better *a priori* experimental agreement (*χ*^2^ = 1.8, though only for a subset of RDCs included in calculations above) (57). Thus, while issues remain with RNA force fields (74), our results emphasize the importance of sampling slow degrees of freedom when making direct comparisons to experiment. The success of the coarse-grained model in reproducing experimental RDCs also motivates even further development of techniques that accelerate sampling within an all-atom framework.

In the approach used here, WExplore only directly accelerates sampling of different interhelical conformations. However, we find that it also significantly accelerates sampling of some orthogonal degrees of freedom, such as the breaking and forming base pair A22-U40. Our configuration space network analysis shows that this base pair serves as a gate that separates the conformational landscape into a lower entropy ‘closed’ partition of states and a high entropy ‘open’ set of states that are characterized by high and low values of the total twist, respectively. This increase in entropy primarily comes from allowing the molecule to access conformations that would be otherwise disallowed by steric and connectivity constraints posed by a formed A22-U40 pair. Experimental studies have indicated that instability of the A22-U40 pair plays a central role in promoting TAR interhelical bending, though whether this directly arises from base pair breaking or weak stacking with the upper helix has been unclear (13,28). Our results suggest a new mechanism, where in addition to destabilizing interhelical stacking, A22-U40 melting plays a direct role, allowing the molecule to access new conformations that are either highly bent, highly twisted or both. Interestingly, prior observations from a survey of internal loop motifs in the Protein Data Bank suggested that tertiary interactions and ligands can stabilize broken pairs within junctions and thus allow the molecule to populate otherwise topologically disallowed interhelical conformations that are important for folding (56). The fact that we find that ‘open’ TAR states are specifically bound by a small molecule ligand here suggests that such high entropy states may be a generally important feature of RNA bulges and internal loops. Significantly, the numerous ‘open’ TAR states we identify also provide a novel set of targets for drug discovery efforts.

While A22-U40 melting is accelerated by WExplore, relaxation is limited along some other orthogonal degrees of freedom. In particular, U23 unstacking from A22 is never observed, whereas it is in simulations that extend for a longer period of continuous simulation time than the effective 14 ns per replica used here. While U23 stacking prevents TAR from adopting coaxially stacked conformations, and thus in theory our method should accelerate its unstacking, the density of unique Euler angle conformations when TAR is coaxially stacked is low, making our method inefficient for this purpose. In contrast, TAR accesses a high density of new Euler angle conformations upon melting of the A22-U40 pair. Notably, when the A22-U40 pair is restrained, sampling of coaxially stacked conformations is significantly enhanced (Supplementary Figure S1). One strategy that could alleviate the slow sampling of bulge degrees of freedom would be to use a more heterogeneous starting pool of trajectories. These could be frames from a long trajectory, or separately initialized copies of the system with different ion positions. Another approach could explicitly combine the statistics of WExplore with a small number of long straightforward trajectories. As demonstrated above, this can result in better agreement with experiment, as it combines the broad sampling in order parameter space provided by WExplore with thorough orthogonal sampling provided by straightforward trajectories.

Overall, our results show the power of using WExplore in conjunction with interhelical Euler angles to accelerate conformational sampling in RNA molecules. While here we apply our method to a two-way junction, the method can be easily extended to study higher-order junctions, such as three-way and four-way junctions that feature prominently in many biologically important RNAs. Here, one could define regions using *n* − 1 sets of Euler angles measured between *n* helices. Previous work has shown that WExplore shows efficiency gains in model systems between 10 and 20 orthogonal order parameters (49), which easily covers both three-way and four-way junctions, having 6 and 9 interhelical angles, respectively. To date, sampling interhelical dynamics of higher-order junctions in an undirected manner on an atomic level has simply been out of reach. We believe simulations exploring such dynamics will provide both greater insight into biological mechanisms, as well as the factors governing RNA dynamics in general.

## AVAILABILITY

Representative structures for each node in the network presented here are available for download from http://brooks.chem.lsa.umich.edu/data.

## SUPPLEMENTARY DATA

Supplementary data are available at NAR Online.

SUPPLEMENTARY DATA
