# Supplementary material for: Efficient in silico exploration of RNA interhelical conformations using Euler angles and WExplore
Source: Nucleic Acids Res. 2014 Oct 7;42(19):12126–37. doi: 10.1093/nar/gku799 (PMC4231733; doi:10.1093/nar/gku799)
Supplement: SUPPLEMENTARY DATA [file supp_42_19_12126__index.html]

Efficient in silico exploration of RNA interhelical conformations using Euler angles and WExplore — SUPPLEMENTARY DATA 

# Efficient *in silico* exploration of RNA interhelical conformations using Euler angles and WExplore

## SUPPLEMENTARY DATA

**Files in this Data Supplement:**

- SUPPLEMENTARY DATA
- SUPPLEMENTARY DATA
