# Supplementary material for: Efficient in silico exploration of RNA interhelical conformations using Euler angles and WExplore
Source: Nucleic Acids Res. 2014 Oct 7;42(19):12126–37. doi: 10.1093/nar/gku799 (PMC4231733; doi:10.1093/nar/gku799)
Supplement: SUPPLEMENTARY DATA [file supp_gku799_nar-01521-r-2014-File001.pdf]

Efficient In-Silico Exploration of RNA Interhelical Conformations Using Euler Angles and WExplore
Supporting Information

Alex Dickson, Anthony M. Mustoe, Loïc Salmon and Charles L. Brooks III

(Dated: July 17, 2014)

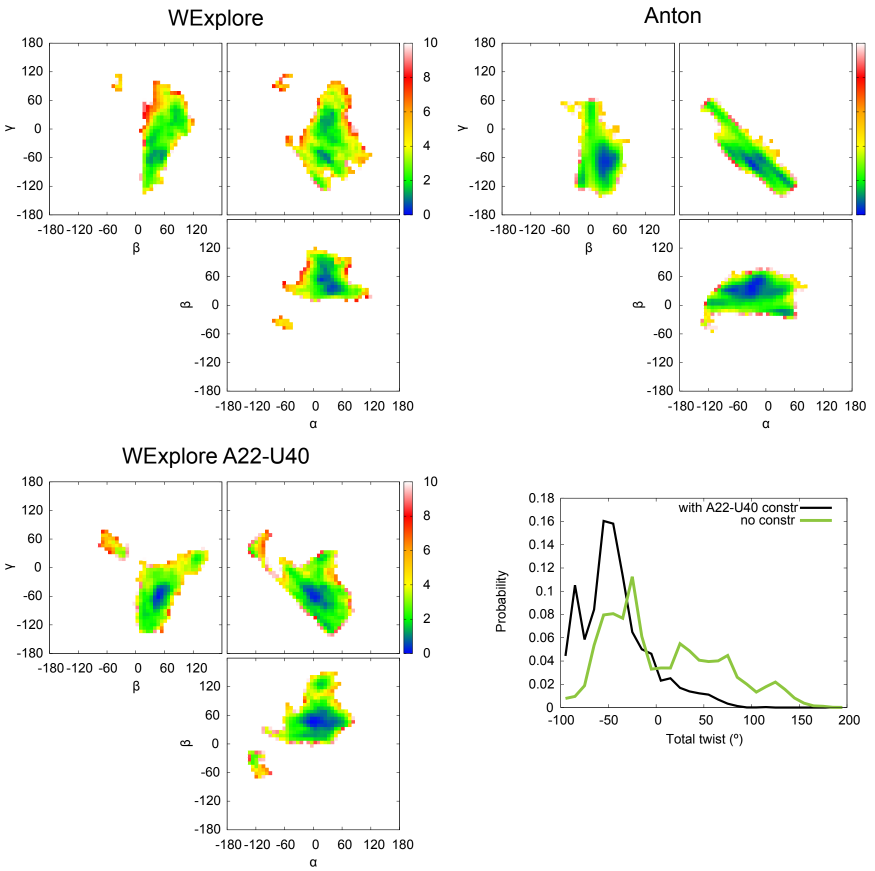

FIG. S1: A comparison of sampling projections onto $\alpha_h - \beta_h$, $\alpha_h - \gamma_h$ and $\beta_h - \gamma_h$ planes. (Top left) Sampling obtained using the WExplore algorithm. The color bar shows free energy in units of kT, and the plots show the sampling distribution after 680 cycles, or a total sampling of 0.65 μs. (Top right) Sampling obtained previously using a long staightforward trajectories on the Anton supercomputer [1]. The color bar shows probability, and the total sampling time was 8.2 μs. (Bottom left) Same as WExplore, except a harmonic restraint is used to keep the A22-U40 base pair intact. (Bottom right) A comparison of the twist values sampled by the constrained (black curve) and unconstrained (green curve) WExplore simulations.

[1] L. Salmon, G. Bascom, I. Andricioaei, and H. M. Al-hashimi, J. Am. Chem. Soc. **135**, 5457 (2013).

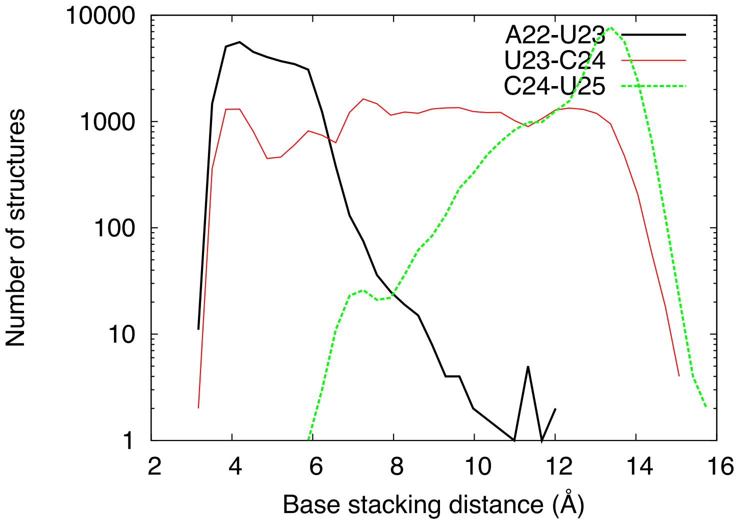

FIG. S2: Distribution of distances between adjacent bulge bases in the WExplore simulation. Distances are calculated using a mass weighted distance between the centers of mass of the bases of each nucleotide. The A22-U23 distance (black curve) shows that the two bases are mostly stacked, with both bases flipped in. C24 samples both flipped-in and -out configurations (red curve). U25 is not observed to interact favorably with C24 (green curve), and remains flipped-out throughout the simulation.

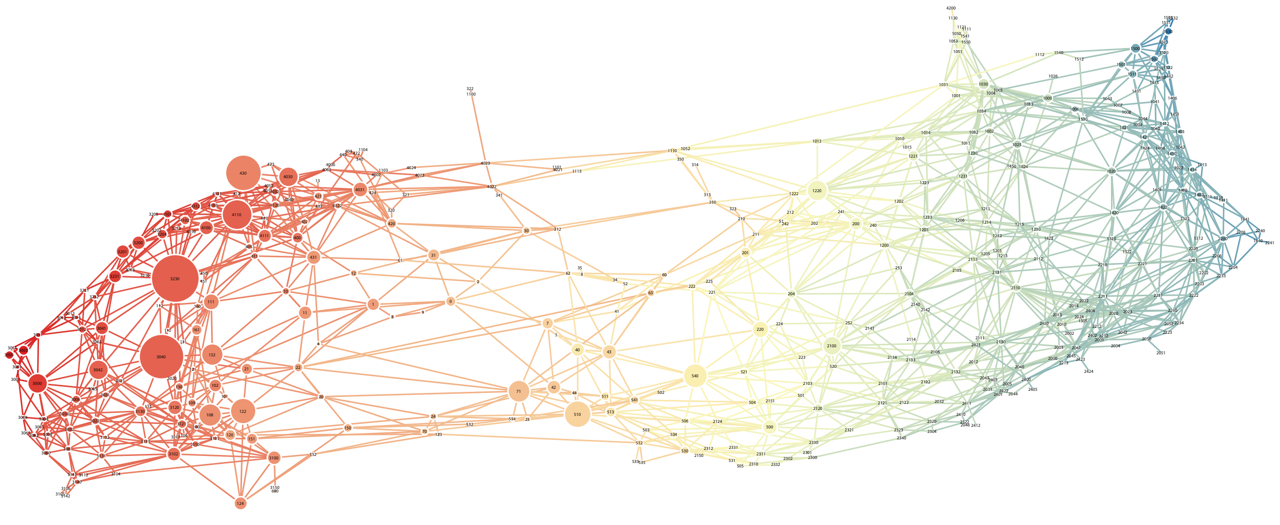

FIG. S3: A configuration space network with node labels, for use with the Supplemental Worksheet 1. Nodes are colored according to the total twist.

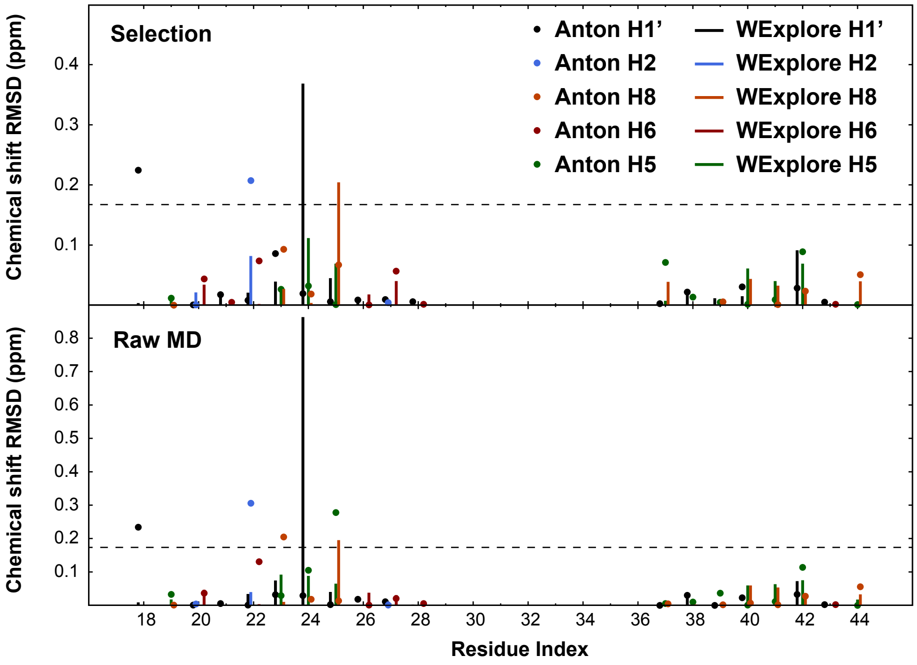

FIG. S4: Agreement of chemical shifts predicted with the WExplore ensemble with experimental values. (Top) The SAS-selected ensemble for both WExplore (bars) and Anton (dots). This is the same SAS ensemble discussed above, which is chosen to optimize agreement with experimental RDC values. The colors indicate the specific side chain atom used to compute the chemical shift. The dashed line approximately indicates the standard error of the NMR chemical shift predictor. (Bottom) The raw ensembles for both WExplore and Anton.

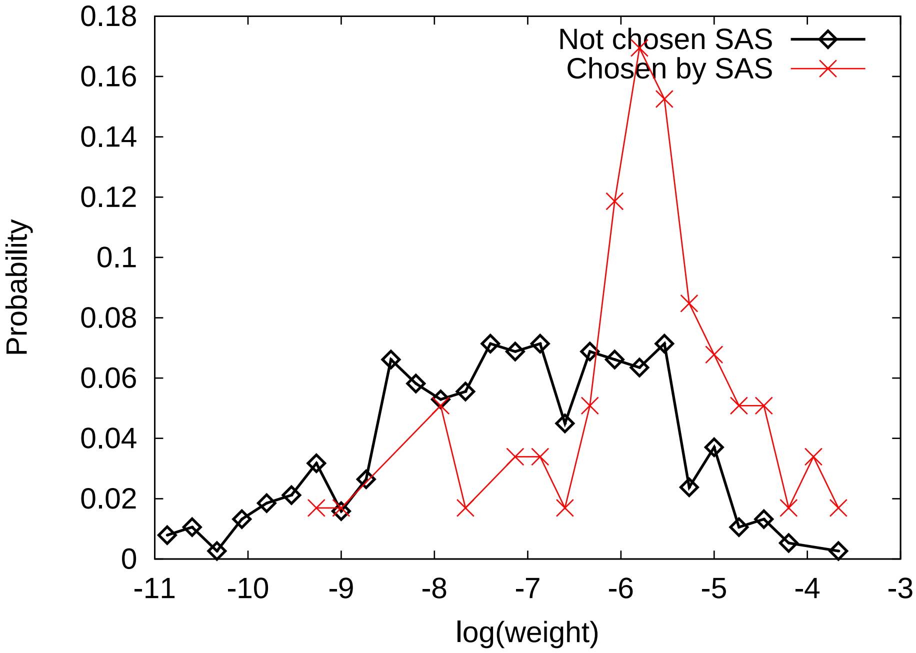

FIG. S5: Comparison of the statistical probabilities (weights) of states that are chosen by the sample and select procedure (red curve) with those that are not (black curve).

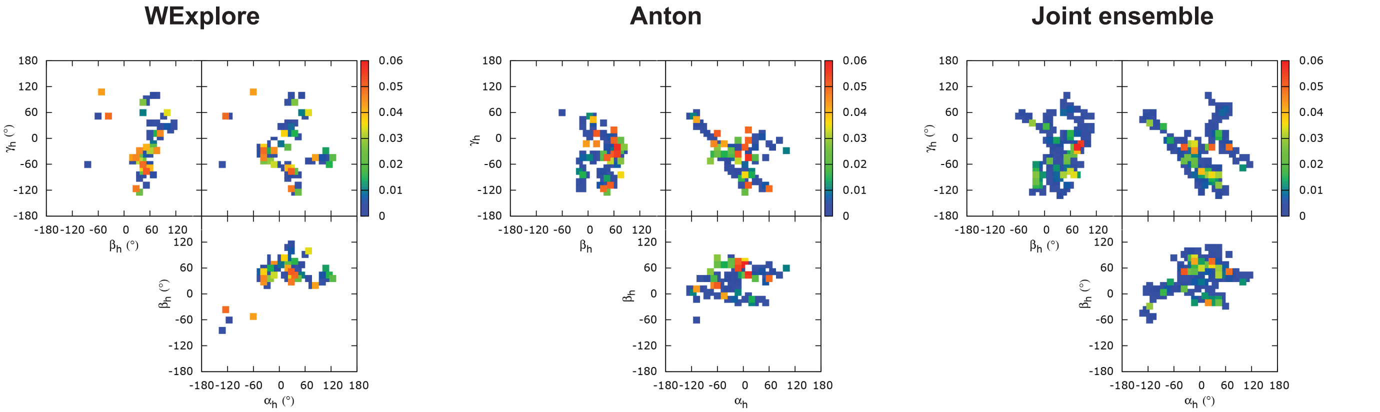

FIG. S6: The probability of being selected as a SAS conformer, projected onto the $\alpha_h - \beta_h$, $\alpha_h - \gamma_h$ and $\beta_h - \gamma_h$ subplanes, for the WExplore (left), Anton (middle) and joint WExplore-Anton (right) ensembles.
